# Perioperative characteristics and short-term morbidity after surgery for renal hyperparathyroidism: multicentre EUROCRINE^®^ registry study

**DOI:** 10.1093/bjsopen/zraf048

**Published:** 2025-06-12

**Authors:** Klaas Van Den Heede, Nele Brusselaers, Martin Almquist, Philipp Riss, Marco Raffaelli, Sam Van Slycke

**Affiliations:** Department of General and Endocrine Surgery, Onze–Lieve–Vrouw Hospital Aalst–Asse–Ninove, Aalst, Belgium; Department of Women’s and Children’s Health, Karolinska Institute, Karolinska Hospital, Stockholm, Sweden; Global Health Institute, University of Antwerp, Wilrijk, Belgium; Department of Public Health and Primary Care, University Hospital Ghent, Ghent, Belgium; Department of Surgery, Skåne University Hospital, Lund, Sweden; Department of Clinical Sciences, Lund University, Lund, Sweden; Department of General Surgery, Division of Visceral Surgery, Section of Endocrine Surgery, University of Vienna, Vienna, Austria; L'Unità Operativa di Chirurgia Endocrina e Metabolica, Fondazione Policlinico Universitario A. Gemelli IRCCS, Rome, Italy; Centro di Ricerca in Chirurgia delle Ghiandole Endocrine e dell’Obesità, Università Cattolica del Sacro Cuore, Rome, Italy; Department of General and Endocrine Surgery, Onze–Lieve–Vrouw Hospital Aalst–Asse–Ninove, Aalst, Belgium; Global Health Institute, University of Antwerp, Wilrijk, Belgium; Department of General Surgery, AZ Damiaan Ostende, Ostend, Belgium

## Abstract

**Background:**

Parathyroid surgery is an appropriate alternative for renal hyperparathyroidism (rHPT) in patients in whom medical therapy fails. European morbidity and outcome data for clearly defined cohorts, potentially reflecting contemporary clinical practice, remain scarce.

**Method:**

Data were extracted from the EUROCRINE^®^ database on all operations for secondary rHPT between 1 January 2015 and 31 December 2021. Multivariable logistic regression analysis was used to identify risk factors for complications. Subgroup analyses were conducted for the two major surgical approaches (subtotal parathyroidectomy or total thyroidectomy with parathyroid transplantation), as well as for redo and concomitant thyroid surgery. The primary outcome was 30-day morbidity.

**Results:**

After excluding 324 patients, data were analysed for 1165 patients, who underwent primary surgery (859), redo surgery (135), or parathyroid surgery with concomitant (planned or unplanned) thyroid surgery (171). The postoperative complication rate was 13.8% (161 patients). Reintervention for bleeding was necessary in 22 patients (1.9%). The length of hospital stay was >1 week in 108 patients (9.8%), and was shorter in the redo parathyroidectomy than first-time parathyroidectomy group (52.0% (66) *versus* 36.6% (299) discharged within 2 days, respectively). No risk factors for complications could be identified in either the overall or subgroup analyses. In the case of redo surgery or primary surgery with concomitant thyroid surgery, recurrent laryngeal nerve palsy (6.7 *versus* 3.5%, respectively), revision surgery for bleeding (2.2 *versus* 1.2%, respectively), and wound infection rates (0.7 *versus* 0.0%, respectively) remained low.

**Conclusion:**

This large European multicentre cohort study demonstrates the safety and low morbidity of parathyroid surgery for rHPT.

## Teaser

This multicentre surgical cohort study confirms the safety and low morbidity of parathyroid surgery for renal hyperparathyroidism in the largest European study population to date.

## Introduction

Chronic kidney disease has become a common condition worldwide, with 10% of the population aged >18 years having at least stage 3 disease (glomerular filtration rate <60 ml/min/1.73 m^2^ for at least 3 months), as defined by the Kidney Disease Outcomes Quality Initiative^1^. It is expected that the prevalence of chronic kidney disease will increase further in the next decade, due to a larger number of patients with chronic hypertension and diabetes in an aging global population^2^. A very common complication of chronic kidney disease is secondary hyperparathyroidism^3^, also referred to as renal hyperparathyroidism (rHPT), which occurs in up to 30% of patients with end-stage renal disease^4^. Secondary hyperparathyroidism occurs early after the onset of renal failure and is caused by decreased phosphorus excretion, decreased vitamin D levels, inadequate vitamin D activation, and hypocalcaemia^3^. The disease-related symptoms often reduce quality of life^5,6^. In addition, rHPT leads to an increased risk of bone fractures, cardiovascular disease, and even mortality^3^. The first-line treatment for rHPT is appropriate medical treatment (vitamin D supplementation, reduction in phosphate intake, and/or calcimimetics). In some cases, rHPT resolves after renal transplantation^3^. However, in 17–50% of patients, rHPT persists more than 1 year after transplantation due to an autonomous hypersecretion of parathyroid hormone (PTH). This is known as tertiary rHPT^4^.

Parathyroid surgery is the first-line treatment for primary hyperparathyroidism and is an appropriate alternative for secondary and tertiary hyperparathyroidism in patients with rHPT in whom medical therapy fails. After 10 years of dialysis without a functional kidney transplant, approximately 15% of patients will need a parathyroidectomy; after 20 years, this proportion rises to 38%^7^. The Kidney Disease Outcomes Quality Initiative guidelines recommend surgery in patients with severe hyperparathyroidism, defined as serum PTH levels >800 pg/ml accompanied by hypercalcaemia and/or hyperphosphataemia and refractory to medical treatment^8^. Parathyroidectomy is considered safe and effective for the treatment of rHPT, and several surgical options are available, including subtotal or total parathyroidectomy with or without autotransplantation of parathyroid tissue, and with or without thymus resection^9^. The choice of surgical approach is not defined by clear guidelines and varies according to patient characteristics, clinical experience, the surgeon’s preference, surgical volume, and hospital volume^7,10^. The choice of surgical procedure will also depend on the intended outcome of the procedure, because the rHPT cohort consists of patients on the kidney transplant list, patients who cannot have a kidney transplant (due to additional co-morbidities), and patients after a kidney transplant (functioning or not).

A recent analysis of the American College of Surgeons’ National Surgical Quality Improvement Program (NSQIP) database evaluated over 21,000 parathyroidectomies performed between 2006 and 2014 (including 2249 parathyroidectomies for rHPT)^11^. The authors of that study reported significantly higher overall 30-day morbidity and mortality following surgery for rHPT than primary hyperparathyroidism^11^. Moreover, a recent comparative analysis from a high-volume centre in the UK stated that morbidity is under-reported, with parathyroidectomy for rHPT associated with higher mortality and higher overall, surgery-related, and systemic-related morbidity than surgery for primary hyperparathyroidism^12^.

Extensive European data on morbidity and short-term outcomes reflecting day-to-day practice in clearly defined cohorts remain scarce in the case of surgical treatment of rHPT. A good understanding of postoperative complications is important to correctly inform patients, avoid litigation, and plan for future healthcare and medical costs.

Therefore, the aim of the present multicentre study was to perform a descriptive, retrospective analysis of a European multicentre cohort to evaluate short-term (30-day) morbidity in patients who underwent surgery for rHPT.

## Methods

### Study cohort

The study protocol and data extraction were approved by the EUROCRINE^®^ board and the Ethics Committee of the Onze–Lieve–Vrouw (OLV) Hospital Aalst–Asse–Ninove (Case number 2021-100). Data from the EUROCRINE^®^ database on all operations for rHPT were extracted on 23 June 2022. All adult patients who underwent surgery for rHPT between 1 January 2015 and 31 December 2021 were eligible for inclusion. Patients with parathyroid surgery after a successful, functional kidney transplant were excluded from the analysis because they may represent a different cohort with differences in surgical strategy, morbidity, and/or outcome. Patients who were not on dialysis but for whom information was lacking as to whether they had received a kidney transplant were also excluded. In addition, patients with illogical values, such as patient discharge before the date of surgery, and patients with a first follow-up more than 12 months after surgery were excluded from the analysis.

The EUROCRINE^®^ database is an online pan-European endocrine surgical quality registry collecting data on all endocrine surgical procedures^13–17^. Quality control occurs at local, national, and international levels, with each participating centre having signed a specific agreement for correct data entry. The EUROCRINE^®^ board members are responsible for assessing compliance at the national level. Data entry is not mandatory. During the study period, 15 clinics entered data for 10 or more patients; 40 clinics entered data for at least 3.

### Patient characteristics and other variables

Information on patient characteristics and other variables was extracted from the database. This included demographic data (age, sex), biochemical data (preoperative and postoperative serum calcium levels, preoperative serum PTH (normal and 1–5-, 6–10-, 11–20-, and > 20-times above normal)), preoperative treatment and imaging data, surgical data, and postoperative data.

Preoperative treatment and imaging data included previous kidney transplant, dialysis treatment, localization imaging studies, and preoperative and postoperative indirect/direct laryngoscopy. Surgical data included the type of surgeon (consultant, trainee), the type and duration of the parathyroid surgery, the number of transplanted parathyroid glands, the number of parathyroid glands identified and excised, thymus operation, thoracic exploration, concomitant planned/unplanned thyroid operations, frozen section, intraoperative PTH (ioPTH), and the use of intraoperative nerve monitoring (IONM).

Postoperative data included revision for haemorrhage, the length of hospital stay (defined as the number of days in hospital after surgery: 0–2, 3–4, and >4 days), 30-day morbidity (suspected damage to the external branch of the superior laryngeal nerve (EBSLN), recurrent laryngeal nerve (RLN) palsy, and wound infection), 30-day all-cause mortality, calcium status at first follow-up (hypocalcaemia with and without treatment, normocalcaemia, and hypercalcaemia), reoperation for rHPT, and pathology of resected specimens.

### Definitions

For the descriptive analysis, three subgroups were considered: first-time ‘standard’ parathyroidectomy, re-operative (redo) parathyroidectomy, and parathyroidectomy with concomitant thyroid surgery. The database did not enable us to distinguish between planned and unplanned concomitant thyroid surgery. The primary outcome was 30-day morbidity. This was defined as the presence of one or more of the following complications: reintervention for haemorrhage, wound infection, RLN palsy, or any other complication that would be classified at least Clavien–Dindo grade II^18^, as well as a length of hospital stay >10 days after surgery. A minimum follow-up of 30 days after surgery was required for inclusion in the analysis. Because hypocalcaemia is to be expected in most patients, a prolonged length of hospital stay of >10 days was chosen in this study as a surrogate for the most severe hypocalcaemia/hungry bone syndrome, because no data were available regarding the evolution and treatment of postoperative hypocalcaemia. The use of ioPTH was defined as performing blood tests during surgery to confirm an ‘adequate’ drop in PTH. However, how this translates in the case of subtotal or total parathyroidectomy for rHPT is not defined, because the published criteria apply to surgery for primary HPT. A ‘correct’ ioPTH would be defined as a sufficient drop (>50%) in PTH immediately after surgery leading to a continued drop in the hours after surgery. For subgroup analysis, subtotal parathyroidectomy was defined as the removal of 3.5 parathyroid glands after identifying all 4 glands. Total parathyroidectomy was defined as the removal of all four parathyroid glands with reimplantation of parathyroid tissue. All procedures with concomitant thyroid surgery, redo surgery, or less than subtotal parathyroidectomies were excluded from this subgroup analysis. The redo surgery cohort reflects previous parathyroid and previous thyroid surgery.

### Statistical analysis

The normality of data distribution was evaluated using the Shapiro–Wilk test. Continuous variables are reported as the median with interquartile range (i.q.r.), whereas categorical variables are presented as counts and percentages. Descriptive statistics were used to compare patients who underwent first-time surgery for rHPT, redo surgery for rHPT, or parathyroid surgery with concomitant planned/unplanned thyroid surgery using χ^2^ tests and Kruskal–Wallis tests, as appropriate. To identify associations between important surgical morbidity with preoperative and postoperative variables, univariable and multivariable logistic regression analyses were conducted to generate odds ratios (ORs). For the logistic model, ORs and 95% confidence intervals are reported. All statistical analyses were conducted using STATA^®^ (V.16.1/MP; StataCorp, College Station, TX, USA).

## Results

**Fig. 1:**
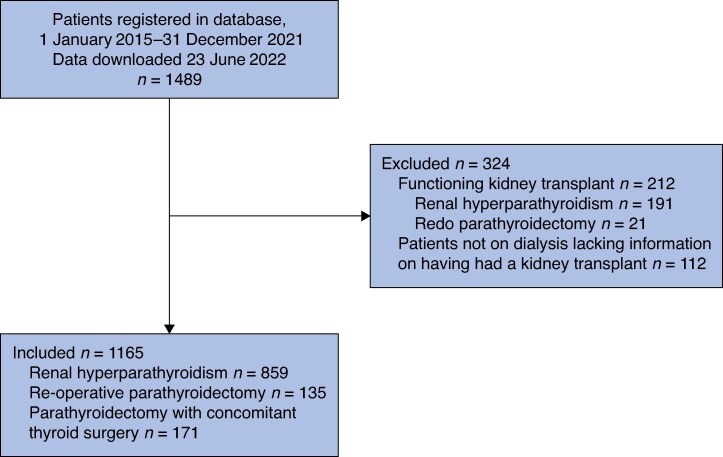
Study flow chart

After excluding 324 patients based on the exclusion criteria, 1165 patients from 12 countries and 66 clinics who underwent parathyroid surgery for rHPT without a functional kidney transplant were included in the analysis (*Fig. 1*). The median age of the total cohort was 53 (i.q.r. 42–61) years, and an equal number of male and female patients underwent parathyroid surgery (female-to-male ratio 0.99). The median total serum calcium before surgery was 2.30 (i.q.r. 2.12–2.48) mmol/L and the preoperative PTH was at least 11 times above the normal range in approximately three-quarters of patients (885, 75.9%).

### Preoperative characteristics

In all, 859 patients (73.7%) underwent first-time ‘standard’ parathyroid surgery. Planned or unplanned concomitant thyroid surgery was performed in 171 patients (14.7%): 40 total thyroidectomies (23.4%), 96 lobectomies (56.1%), 3 isthmectomies (1.8%), and 32 partial lobectomies (18.7%). Redo parathyroid surgery was performed in 135 patients (11.6%). Median patient age and the female-to-male ratio were higher in the subgroup undergoing concomitant thyroid surgery than in the first-time and redo parathyroid surgery subgroups (57 *versus* 52 and 53 years [*P <* 0.001] and 1.44 *versus* 0.93 and 0.96 [*P* = 0.033], respectively). Preoperative PTH was significantly lower in the redo parathyroid surgery subgroup. In the redo parathyroid surgery subgroup, 41 patients (30.4%) had undergone prior thyroid surgery. In addition, a higher number of patients in the redo surgery subgroup (20, 14.8%) had a failed kidney transplant than in the first-time parathyroid surgery (7.9%) and parathyroid surgery with concomitant thyroid surgery (8.8%) groups (*P =* 0.032). Localization imaging was performed before surgery in approximately three-quarters of patients in the redo parathyroid surgery subgroup (100, 77.5%), which was significantly higher than in the first-time parathyroid surgery, with or without concomitant thyroid surgery, group (594, 57.7%; *P <* 0.001). Approximately 80% of the total cohort underwent preoperative laryngoscopy, diagnosing five preoperative RLN paralyses (0.4%) before redo surgery (*Table 1*).

**Table 1:** Preoperative characteristics of the groups undergoing primary surgery for renal hyperparathyroidism, redo surgery, and primary surgery with concomitant thyroid surgery

	Primary surgery (*n* = 859; 73.7%)	Redo surgery (*n* = 135; 11.6%)	Primary surgery+ thyroid surgery (*n* = 171; 14.7%)	P*
**Sex**				
Female-to-male ratio	0.93	0.96	1.44	0.033
**Age (years), median (i.q.r.)**	52 (41–60)	53 (44–59)	57 (48–63)	
<46	296 (34.5%)	38 (28.1%)	35 (20.5%)	<0.001
46–59	335 (39.0%)	66 (48.9%)	70 (40.9%)	
>59	228 (26.5%)	31 (23.0%)	66 (38.6%)	
**Biochemistry**				
Total serum calcium (mmol/L), median (i.q.r.)	2.30 (2.13–2.46)	2.26 (2.10–2.46)	2.30 (2.14–2.53)	
Missing data	95 (11.1%)	19 (14.1%)	16 (9.4%)	
Serum PTH				
Within normal range	4 (0.5%)	1 (0.7%)	0 (0.0%)	<0.001
1–5× above normal range	46 (5.4%)	10 (7.4%)	8 (4.7%)	
6–10× above normal range	128 (14.9%)	36 (26.7%)	41 (24.0%)	
11–20× above normal range	318 (37.0%)	52 (38.5%)	64 (37.4%)	
>20× above normal range	358 (41.7%)	35 (25.9%)	58 (33.9%)	
Missing data	5 (0.6%)	1 (0.7%)	0 (0.0%)	
**Previous thyroid surgery**				
Yes	0 (0.0%)	41 (30.4%)	0 (0.0%)	
**Treatment of kidney disease**				
Dialysis patient	791 (92.1%)	115 (85.2%)	156 (91.2%)	0.032
Dialysis patient, transplanted	68 (7.9%)	20 (14.8%)	15 (8.8%)	
**Localization examination**				
Yes	508 (61.3%)	100 (77.5%)	86 (51.8%)	<0.001
No	321 (38.7%)	29 (22.5%)	80 (48.2%)	
Missing data	30 (3.5%)	6 (4.4%)	5 (2.9%)	
**Preoperative indirect/direct laryngoscopy**				
Normal	695 (80.9%)	108 (80.0%)	135 (78.9%)	0.763
Not performed	161 (18.7%)	24 (17.8%)	36 (21.1%)	
Paresis of the recurrent laryngeal nerve				
Right	1 (0.1%)	3 (2.2%)	0 (0.0%)	
Left	2 (0.2%)	0 (0.0%)	0 (0.0%)	
Bilateral	0 (0.0%)	0 (0.0%)	0 (0.0%)	

Values are *n* (%) unless otherwise stated. Kruskal–Wallis test was used to generate *P* values. i.q.r., interquartile range; PTH, parathyroid hormone.

### Surgical characteristics

Most procedures were performed by senior consultants (907, 92.5%). The duration of surgery was twice as long in the subgroup with concomitant thyroid surgery (119 min) than in the other two groups. A single gland was excised in approximately half the patients undergoing redo parathyroid surgery (71, 52.6%), and no gland was identified or removed in eight patients (5.9%). In the total cohort, 15 patients (1.7%) had supernumerary parathyroid glands. In the group undergoing first-time parathyroid surgery without concomitant thyroid surgery, most patients (537, 62.5%) underwent subtotal parathyroidectomy with (170, 31.7%) or without (367, 68.3%) thymus resection. Two thoracoscopic parathyroid resections (0.2%) were performed in the first-time parathyroidectomy group and two sternotomies (1.5%) were performed in the redo parathyroid surgery group. IONM was used in 40.3% of patients in the first-time surgery cohort, in 42.0% of patients in the redo surgery cohort, and in 59.4% of patients undergoing parathyroid surgery with concomitant thyroid surgery. In 28 patients (2.4%), a visible lesion of the RLN or persistent loss of signal on neuromonitoring was noted during surgery. Frozen section was used in 250 patients (29.1%), mainly in those undergoing concomitant thyroid surgery, and was correct in 247 patients (98.8%). In all, 407 patients (47.4%) underwent ioPTH measurements, predominantly in the undergoing redo surgery group, and these measurements were considered correct in 365 patients (89.7%) (*Table 2*).

**Table 2:** Surgical characteristics of the groups undergoing primary surgery for renal hyperparathyroidism, redo surgery, and primary surgery with concomitant thyroid surgery

	Primary surgery (*n* = 859; 73.7%)	Redo surgery (*n* = 135; 11.6%)	Primary surgery+ thyroid surgery (*n* = 171; 14.7%)	P*
**Surgeon**				
Consultant	681 (93.7%)	97 (92.4%)	129 (87.2%)	0.023
Trainee	46 (6.3%)	8 (7.6%)	19 (12.8%)	
Missing data	132 (15.4%)	30 (22.2%)	23 (13.5%)	
**Duration of surgery (min), median (i.q.r.)**	64 (40–100)	63 (40–105)	119 (86–155)	
<56	342 (41.7%)	55 (43.7%)	11 (6.6%)	<0.001
56–100	278 (33.9%)	36 (28.6%)	45 (27.1%)	
>100	201 (24.5%)	35 (27.8%)	110 (66.3%)	
Missing data	38 (4.4%)	9 (6.7%)	5 (2.9%)	
**Parathyroid operation**				
Less than subtotal parathyroidectomy	60 (7.0%)	81 (60.0%)	31 (18.1%)	
Single gland	15 (1.7%)	71 (52.6%)	4 (2.3%)	
Subtotal parathyroidectomy	537 (62.5%)	N/A	102 (59.6%)	
Total parathyroidectomy	262 (30.5%)	N/A	38 (22.2%)	
**No. of glands identified**				
0	0 (0.0%)	8 (5.9%)	0 (0.0%)	<0.001
1	9 (1.0%)	70 (51.9%)	4 (2.3%)	
2	16 (1.9%)	34 (25.2%)	10 (5.8%)	
3	52 (6.1%)	12 (8.9%)	33 (19.3%)	
4	769 (89.5%)	10 (7.4%)	123 (71.9%)	
>4	13 (1.5%)	1 (0.7%)	1 (0.6%)	
**Thymus operation**				
No	627 (73.9%)	112 (83.0%)	98 (57.6%)	<0.001
Biopsy/partial resection of thymus	10 (1.2%)	2 (1.5%)	3 (1.8%)	
Thymus resection	212 (25.0%)	21 (15.6%)	69 (40.6%)	
**Thoracic exploration**				
No	856 (99.7%)	133 (98.5%)	171 (100.0%)	
Thoracoscopy	2 (0.2%)	0 (0.0%)	0 (0.0%)	
Sternotomy	1 (0.1%)	2 (1.5%)	0 (0.0%)	
**Thyroid operation**				
No	859 (100.0%)	117 (86.7%)	0 (0.0%)	
Total thyroidectomy	0 (0.0%)	0 (0.0%)	40 (23.4%)	
Unilateral lobectomy of thyroid gland	0 (0.0%)	15 (11.1%)	96 (56.1%)	
Other (less than lobectomy)	0 (0.0%)	3 (2.2%)	35 (20.5%)	
**Recurrent laryngeal nerve**				
ID right RLN	692 (80.6%)	66 (48.9%)	156 (91.2%)	0.995
ID left RLN	690 (80.3%)	67 (49.6%)	156 (91.2%)	
RLN damage noted during surgery				
Bilateral	5 (0.6%)	1 (0.7%)	0 (0.0%)	0.006
Left	4 (0.5%)	1 (0.7%)	3 (1.8%)	
Right	7 (0.8%)	0 (0.0%)	7 (4.1%)	
No damage	843 (98.1%)	133 (98.5%)	161 (94.2%)	
**Frozen section**				
Correct	152 (17.7%)	28 (20.7%)	67 (39.2%)	0.435
Misleading	1 (0.1%)	1 (0.7%)	1 (0.6%)	
Not used	706 (82.2%)	106 (78.5%)	103 (60.2%)	
**Intraoperative PTH**				
Correct	252 (29.3%)	52 (38.5%)	61 (35.7%)	0.128
Misleading	25 (2.9%)	11 (8.1%)	6 (3.5%)	
Not used	582 (67.8%)	72 (53.3%)	104 (60.8%)	
**Intraoperative nerve monitoring**				
Yes	64 (40.3%)	13 (42.0%)	19 (59.4%)	0.136
Continuous	2 (3.1%)	2 (15.4%)	1 (5.3%)	
Intermittent	62 (96.9%)	11 (84.6%)	18 (94.7%)	
No	95 (59.7%)	18 (58.1%)	13 (40.6%)	
Missing data	700 (81.5%)	104 (77.0%)	139 (81.3%)	

Values are *n* (%) unless otherwise stated. Kruskal–Wallis test was used to generate *P* values. i.q.r., interquartile range; ID, identification; RLN, recurrent laryngeal nerve; PTH, parathyroid hormone.

### Postoperative outcomes

Postoperative complications occurred in 161 patients (13.8%). Reintervention for bleeding was necessary in 22 patients (2.6%). The length of hospital stay was >10 days in 47 patients (4.0%), and was shorter in the redo parathyroidectomy group, because over half these patients were discharged within 2 days after surgery (66, 52.0%) compared with around one-third of patients in the first-time parathyroidectomy without thyroid surgery group (299, 36.6%). After a median follow-up of eight (i.q.r. 4–15) weeks, there were 8 wound infections (0.3%), 30 RLN palsies (2.6%), and 7 deaths (0.6%) within the first month after surgery. Even in the redo surgery setting or in the case of concomitant thyroid surgery, rates of RLN palsy (6.7 and 3.5%, respectively), revision surgery for bleeding (2.2 and 1.2%, respectively), and wound infection rates (0.7 *versus* 0.0%, respectively) remained low. Hypocalcaemia was found in 929 (85.1%) patients, 825 with (88.8%) and 104 without (11.2%) treatment. Of the 749 patients (64.3%) with long-term (>1 year) follow-up, 26 (9.4%) underwent reoperation for rHPT. The seven registered deaths within the first month after surgery (0.6%) included two deaths due to myocardial infarction, one due to pulmonary embolism, and one due to pneumonia; the cause of death was missing for the remaining four patients (*Table 3*).

**Table 3:** Postoperative characteristics of the groups undergoing primary surgery for renal hyperparathyroidism, redo surgery, and primary surgery with concomitant thyroid surgery

	Primary surgery (*n* = 859; 73.7%)	Redo surgery (*n* = 135; 11.6%)	Primary surgery + thyroid surgery (*n* = 171; 14.7%)	P*
Haemorrhage	17 (2.0%)	3 (2.2%)	2 (1.2%)	
**Postoperative indirect/direct laryngoscopy**				
Not performed	270 (31.4%)	40 (29.6%)	60 (35.1%)	0.038
Normal	572 (66.6%)	86 (63.7%)	105 (61.4%)	
Paresis of the right RLN	10 (1.2%)	5 (3.7%)	0 (0.0%)	
Paresis of the left RLN	6 (0.7%)	4 (3.0%)	6 (3.5%)	
Bilateral paresis RLN	1 (0.1%)	0 (0.0%)	0 (0.0%)	
**Length of hospital stay (days), median (i.q.r.)**	3 (2–6)	2 (2–4)	4 (2–5)	
0–2	299 (36.6%)	66 (52.0%)	52 (32.1%)	<0.001
3–4	203 (24.8%)	33 (26.0%)	53 (32.7%)	
>4	315 (38.6%)	28 (22.0%)	57 (35.2%)	
Missing data	42 (4.9%)	8 (5.9%)	9 (5.3%)	
30-day all-cause mortality	2 (0.2%)	1 (0.7%)	4 (2.3%)	
**Wound infection**	7 (0.8%)	1 (0.7%)	0 (0.0%)	
Signs of EBSLN damage	11 (1.4%)	0 (0.0%)	1 (0.6%)	
Missing data	47 (5.5%)	8 (5.9%)	10 (5.8%)	
**Indirect/direct laryngoscopy at first follow-up**				
Not performed	261 (30.4%)	43 (31.9%)	63 (36.8%)	0.001
Normal	583 (67.9%)	82 (60.7%)	103 (60.2%)	
Paresis of the right RLN	10 (1.2%)	6 (4.4%)	2 (1.2%)	
Paresis of the left RLN	5 (0.6%)	4 (3.0%)	3 (1.8%)	
Bilateral paresis RLN	0 (0.0%)	0 (0.0%)	0 (0.0%)	
**Calcium status**				
Hypocalcaemia without treatment	73 (8.5%)	17 (12.6%)	14 (8.2%)	0.673
Hypercalcaemia	9 (1.0%)	2 (1.5%)	1 (0.6%)	
Normocalcaemia	108 (12.6%)	20 (14.8%)	22 (12.9%)	
Treatment with calcium and/or vitamin D	617 (71.8%)	88 (65.2%)	120 (70.2%)	
Missing data	52 (6.1%)	8 (5.9%)	14 (8.2%)	
**Total serum calcium (mmol/L), median (i.q.r.)**	2.20 (1.79–2.19)	2.09 (1.87–2.32)	2.03 (1.84–2.16)	
Missing data	641 (74.6%)	90 (66.7%)	93 (54.4%)	
Overall important morbidity	116 (13.5%)	22 (16.3%)	23 (13.4%)	
**Reoperated for sHPT**				
No	511 (59.5%)	78 (57.8%)	134 (78.4%)	0.003
Yes	17 (2.0%)	8 (5.9%)	1 (0.6%)	
Missing data	331 (38.5%)	49 (36.3%)	36 (21.1%)	
Deceased (all-cause mortality)	3 (0.3%)	3 (2.2%)	4 (2.3%)	

Values are *n* (%) unless otherwise stated. Kruskal–Wallis test was used to generate *P* values. RLN, recurrent laryngeal nerve; i.q.r., interquartile range; EBSLN, external branch of the superior laryngeal nerve; sHPT, secondary hyperparathyroidism.

### Pathology

Most patients (920, 79.0%) in the first-time parathyroidectomy groups with and without concomitant thyroid surgery had parathyroid hyperplasia, including 307 with diffuse hyperplasia (33.4%) and 590 with nodular hyperplasia (64.1%). Overall, 183 adenomas (15.7%) were found, including 9 atypical adenomas (4.9%). In the total cohort, five parathyroid cancers (0.4%) were found. A significantly (*P <* 0.001) higher proportion of parathyroid adenomas was found in the redo surgery group (40, 29.6%) than in the first-time surgery group (*Table S1*).

In the overall cohort, univariable logistic regression analysis to identify risk factors for important morbidity (excluding hypocalcaemia) revealed that total parathyroidectomy with transplanted parathyroid (OR 2.33, 95% confidence interval (c.i.) 1.10 to 4.92) was a risk factor for complications and that performing localization imaging (OR 0.67, 95% c.i. 0.47 to 0.96) was protective against complications. This was not confirmed in the multivariable analysis (*Table S2*).

### Risk factors for severe morbidity after subtotal or total parathyroidectomy with transplantation

In the overall cohort, most patients (643, 55.2%) underwent a subtotal (removal of 3.5 parathyroid glands; 395) or total (248) parathyroidectomy with parathyroid transplantation without any concomitant thyroid surgery or previous (para)thyroid surgery (*Table S3*). These homogeneous cohorts were also compared for severe morbidity. The group that underwent subtotal parathyroidectomy had lower preoperative PTH values (*P* = 0.010), less preoperative localization imaging (*P* < 0.001), tended to have a longer surgical time (65 *versus* 50 min; *P* > 0.001), more thymus resections (29.4 *versus* 14.9%; *P* < 0.001), less use of frozen section (15.9 *versus* 25.0%; *P* = 0.007), more revision surgery for bleeding (1.8 *versus* 1.2%; *P* = 0.038), and faster time to discharge (3 *versus* 5 days; *P* < 0.001) than the group that underwent total parathyroidectomy.

Multivariable logistic regression analysis to identify risk factors for important morbidity showed no significant risk factors, besides preoperative PTH values 11 and 20 times above the normal range being protective compared with lower or higher preoperative values (OR 0.40, 95% c.i. 0.18 to 0.82) (*Table 4*).

**Table 4:** Statistical analysis of morbidity risk: subgroup analysis comparing first time surgery with total parathyroidectomy *versus* subtotal parathyroidectomy

	Multivariable logistic regression	
	Odds ratio	*P*
**Sex**		
Female	1 (Reference)	
Male	1.56 (0.81, 3.06)	0.190
Age	0.99 (0.97, 1.01)	0.471
Preoperative serum calcium	1.31 (0.45, 3.82)	0.621
**Preoperative PTH**		
≥20× above normal range	1 (Reference)	
1–5× above normal range	0.38 (0.02, 1.96)	0.352
6–10× above normal range	0.40 (0.18, 0.82)	0.017
11–<20× above normal range	0.41 (0.12, 1.09)	0.106
Within normal range	0.00	0.985
Duration of surgery	1.00 (0.99, 1.01)	0.573
Type of parathyroid surgery*		
Subtotal	1 (Reference)	
Total + parathyroid transplant	1.69 (0.89, 3.22)	0.108
**Thymus operation**		
No	1 (Reference)	
Yes	1.47 (0.66, 3.15)	0.329

Values in parentheses are 95% confidence intervals. *Subtotal parathyroidectomy was defined as the removal of 3.5 parathyroid (PT) glands after identifying all 4 glands. Total parathyroidectomy was defined as the removal of all four PT glands with re-implantation of PT tissue. PTH, parathyroid hormone.

## Discussion

This multicentre retrospective surgical cohort study analysed current surgical practice and short-term outcomes in European patients undergoing surgery for rHPT. The analysis of 1165 patients showed excellent surgical results, with low morbidity and low 30-day all-cause mortality. Overall short-term morbidity was 13.5%. In the overall cohort, the bleeding risk, the immediate RLN palsy risk, the rate of hospital stay >4 days, and the 30-day mortality were low. No clear risk factors for severe morbidity could be identified in multivariable analysis in either the overall or in the subgroups of subtotal parathyroidectomy and total thyroidectomy with parathyroid transplantation. Even in the case of redo surgery or concomitant thyroid surgery, RLN palsy, revision surgery for bleeding, and wound infection rates remained low. Very high preoperative PTH (>20-times normal values) may be a risk factor for morbidity.

To the best of the authors’ knowledge, this is one of the largest multicentre data collections of surgery for rHPT and the largest European multicentre analysis of a recent surgical cohort based on a European quality registry. The choice to exclude patients after successful kidney transplantation and to perform a subgroup analysis of the two most performed surgical treatments strengthen the clinical value of the results and reflect day-to-day practice in high-volume European endocrine surgical centres. Moreover, this is the largest study analysing redo surgery for rHPT. Data collection on an international scale allows for comparisons with peers. If data collection could be made mandatory or automated, it could enable the identification of true surgical treatment patterns, international differences, and surgical complication rates. Medical researchers are well positioned to use databases to answer relevant questions. EUROCRINE^®^ and other clinical databases help gather knowledge on the natural history of disease, outcomes of specific patient populations, knowledge on rare diseases, the effectiveness of medical and surgical interventions, and their interplay in modern healthcare.

In the overall cohort, 57.6% of patients underwent localization imaging studies, even in the case of first-time surgery. The data did not allow specification of which imaging study was performed. An ultrasound could have been the most-performed imaging study to exclude thyroid pathology. The value of ^18^F-fluorocholine positron emission tomography (PET) or dual-phase ^99m^Tc-sestamibi single photon emission computed tomography/computed tomography (CT) may be questioned, because four-gland exploration is the standard in first-time parathyroid surgery for rHPT. However, such imaging modalities may reveal supernumerary glands or extracervical disease prior to surgery^19,20^. Particular in redo surgery, ^18^F-fluorocholine PET/CT could demonstrate better accuracy than conventional imaging modalities in patients with secondary or tertiary hyperparathyroidism. The combination of ^18^F-fluorocholine PET/CT and neck ultrasound may enable better surgical planning. In the present cohort, 1.3% of patients were found to have a supernumerary number of parathyroid glands. Because multi-gland disease is to be expected, the authors do not encourage routine localization imaging other than a neck ultrasound to exclude thyroid pathology.

Approximately 80% of patients underwent a preoperative laryngoscopy, with discovery of 0.3% and 2.2% unknown RLN palsies in the first-time surgery and redo surgery cohorts, respectively. IONM was used in less than half the patients, confirming the results of a recent study by Conroy *et al*. that found endocrine surgeons use IONM selectively^21^. Surgeons who routinely use IONM during thyroidectomy are more likely to use it during parathyroidectomy. Future studies should determine which patients may benefit most from IONM in parathyroidectomy.

Both subtotal parathyroidectomy and total parathyroidectomy with parathyroid autotransplantation are performed in Europe. Surgical outcomes have been comparable and the optimal surgical approach for parathyroidectomy in patients with secondary hyperparathyroidism remains controversial^22^. In the subgroup analysis in the present study, no significant differences in morbidity were noted between the two techniques. This confirms previous findings of similar rates of complications, readmission, and 30-day mortality for subtotal parathyroidectomy and total parathyroidectomy with parathyroid autotransplantation^23^. Subtotal parathyroidectomy was less likely to have an extended hospital stay (3 *versus* 5 days; *P* < 0.001), as found in a previous large American registry^23^.

In the present study, the redo surgery cohort reflected previous parathyroid and previous thyroid surgery. Morbidity in this cohort appears to be comparable to the first-time surgery cohort with equal RLN palsy, bleeding, and wound infection rates. The redo surgery cohort had less hyperplasia and more adenomas than the first-time surgery cohort. Failure to cure a patient should not mean refraining from redo surgery. This had also been stated in previous smaller cohorts^24,25^. Frozen section was most used in the group undergoing parathyroidectomy with concomitant thyroid surgery. This could reflect difficult procedures when not identifying all four glands and performing an unplanned hemithyroidectomy. Five patients with normal preoperative PTH values underwent surgery. Normal PTH levels do not exclude primary or secondary hyperparathyroidism as the cause of hypercalcaemia. In the case of end-organ damage, surgery may be justified. Other possible explanations would be incorrect data or the presence of primary hyperparathyroidism in a patient on dialysis.

The authors acknowledge some limitations of this study. A registry like EUROCRINE^®^ is prone to typing and coding errors, as well as missing data. For example, no data were available on whether concomitant thyroid surgery was planned or not. No information was available about the indication for surgery (what criteria were followed to propose a surgical intervention), and postoperative short- and long-term PTH values were not available. This could potentially lead to a selection bias because higher PTH prior to surgery or larger drops in PTH may lead to a prolonged hospital stay due to a more severe hungry bone syndrome. Moreover, the length of the hospital stay may be affected by differences in healthcare reimbursement among participating European countries, especially in patients staying only a few days. Because reporting in EUROCRINE^®^ is not mandatory, a selection bias may have occurred towards high-volume specialized centres. Only in Sweden is surgical registration mandatory, potentially meaning the results are more representative of northern European practice rather than the entire European area. However, data input from Sweden was not over-represented in the overall cohort.

## Supplementary Material

zraf048_Supplementary_Data

## Data Availability

The data underlying this manuscript will be shared upon reasonable request to the corresponding author.
