# Supplementary material for: Perioperative characteristics and short-term morbidity after surgery for renal hyperparathyroidism: multicentre EUROCRINE® registry study
Source: BJS Open. 2025 Jun 12;9(3):zraf048. doi: 10.1093/bjsopen/zraf048 (PMC12159728; doi:10.1093/bjsopen/zraf048)
Supplement: zraf048_Supplementary_Data [file zraf048_supplementary_data.docx]

**Perioperative characteristics and short-term morbidity after surgery for renal hyperparathyroidism: multicentre EUROCRINE^®^ registry study**

*Klaas Van Den Heede, MD^1^, Nele Brusselaers, MD, PhD^2,3,4^, Martin Almquist, MD, PhD^5,6^, Philipp Riss, MD, PhD^7^, Marco Raffaelli, MD, PhD^8,9^, Sam Van Slycke, MD, PhD^1,3,10^ on behalf of the EUROCRINE^®^ Council*

1. *Department of General and Endocrine Surgery, Onze-Lieve-Vrouw (OLV) Hospital Aalst-Asse-Ninove, Aalst, Belgium*
2. *Department of Women’s and Children’s Health, Karolinska Institute, Karolinska Hospital, Stockholm, Sweden*
3. *Global Health Institute, University of Antwerp, Wilrijk, Belgium*
4. *Department of Public Health and Primary Care, University Hospital Ghent, Ghent, Belgium*
5. *Department of Surgery, Skåne University Hospital, Lund, Sweden*
6. *Department of Clinical Sciences, Lund University, Lund, Sweden*
7. *Department of General Surgery, Division of Visceral Surgery, Section of Endocrine Surgery, University of Vienna, Austria*
8. *U.O. Chirurgia Endocrina e Metabolica, Fondazione Policlinico Universitario A. Gemelli IRCCS, Rome, Italy*
9. *Centro di Ricerca in Chirurgia delle Ghiandole Endocrine e dell’Obesità (C.R.E.O.), Università Cattolica del Sacro Cuore, Rome, Italy*
10. *Department of General Surgery, AZ Damiaan, Ostend, Belgium*

**Corresponding author.** **Corresponding Author:** Klaas Van Den Heede, OLV Hospital Aalst-Asse-Ninove, Moorselbaan 164, 9300 Aalst, Belgium. + 32 53 72 45 06. Email: [klaasvandenheede@hotmail.com](mailto:dr.samvanslycke@gmail.com)

**Supplementary Materials - Index**

| **Supplementary Figures and Tables** |  |
| --- | --- |
| 1. SUPPLEMENTARY TABLE 1. SURGICAL PATHOLOGY of REMOVED GLANDS OF THE OVERALL COHORT | *page 3* |
| 1. SUPPLEMENTARY TABLE 2. STATISTICAL ANALYSIS OF RISK OF MORBIDITY IN THE OVERALL SURGICAL COHORT | *page 4-5* |
| 1. PATIENT CHARACTERISTICS – SUBGROUP ANALYSIS COMPARING FIRST TIME SURGERY WITH TOTAL PARATHYROIDECTOMY VERSUS SUBTOTAL PARATHYROIDECTOMY | *page 6-7* |
|  |  |

**Supplementary Figures and Tables**

SUPPLEMENTARY TABLE 1. SURGICAL PATHOLOGY of REMOVED GLANDS OF THE OVERALL COHORT

|  | rHPT | rHPT Redo | rHPT + Thyroid |  |
| --- | --- | --- | --- | --- |
|  | N = 859 (73.7%) | N = 135 (11.6%) | N = 171 (14.7%) | *P-value* |
| PATHOLOGY |  |  |  |  |
| ° Normal gland | 9 (1.0) | 3 (2.2) | 1 (0.6) | *<.001* |
| ° Parathyroid adenoma | 126 (14.7) | 40 (29.6) | 17 (9.9) |  |
| - Not specified | 109 (86.5) | 30 (75.0) | 13 (76.5) |  |
| - Atypical adenoma | 5 (4.0) | 2 (5.0) | 2 (11.8) |  |
| - Chief cell adenoma | 6 (4.8) | 5 (12.5) | 1 (5.9) |  |
| - Mixed cell adenoma | 0 (0.0) | 1 (0.7) | 0 (0.0) |  |
| - Other | 2 (1.6) | 0 (0.0) | 0 (0.0) |  |
| - Oxyphilic adenoma | 4 (3.2) | 2 (5.0) | 1 (5.9) |  |
| ° Parathyroid cancer | 4 (0.5) | 0 (0.0) | 1 (0.6) |  |
| ° Parathyroid Hyperplasia | 694 (80.8) | 81 (60.0) | 145 (84.8) |  |
| - Diffuse hyperplasia | 231 (33.3) | 25 (30.9) | 51 (35.2) |  |
| - Nodular hyperplasia | 446 (64.3) | 54 (66.7) | 90 (62.1) |  |
| - Unspecified | 17 (2.4) | 2 (2.5) | 4 (2.8) |  |
| ° Uncertain | 1 (0.1) | 2 (1.5) | 0 (0.0) |  |
| *° Missing* | *25 (2.9)* | *9 (6.7)* | *7 (4.1)* |  |

rHPT: renal hyperparathyroidism

SUPPLEMENTARY TABLE 2. STATISTICAL ANALYSIS OF RISK OF MORBIDITY IN THE OVERALL SURGICAL COHORT

|  | Univariable Logistic Regression | | | Multivariable Logistic Regression | |
| --- | --- | --- | --- | --- | --- |
|  | OR | 95% CI |  | OR | 95% CI |
| Sex |  |  |  |  |  |
| ° Female | 1 |  |  | 1 |  |
| ° Male | 0.95 | 0.68-1.33 |  | 0.97 | 0.68-1.39 |
| Age, years |  |  |  |  |  |
| ° <51 | 1 |  |  | 1 |  |
| ° 51-60 | 1.00 | 0.67-1.49 |  | 1.02 | 0.67-1.55 |
| ° >60 | 1.08 | 0.70-1.66 |  | 1.14 | 0.73-1.79 |
| PTH Preop |  |  |  |  |  |
| ° Within normal range | 1 |  |  | 1 |  |
| ° 1-5 times above normal range | 0.33 | 0.03-3.58 |  | 0.28 | 0.02-3.17 |
| ° 6-10 times above normal range | 0.63 | 0.07-5.65 |  | 0.59 | 0.06-5.55 |
| ° 11-20 times above normal range | 0.61 | 0.07-5.31 |  | 0.57 | 0.06-5.27 |
| ° >= 20 times above normal range | 1.18 | 0.14-10.21 |  | 1.07 | 0.12-9.88 |
| Previous thyroid surgery |  |  |  |  |  |
| ° Yes | 1 |  |  | 1 |  |
| ° No | 1.41 | 0.60-3.36 |  | 1.85 | 0.70-4.86 |
| Dialysis |  |  |  |  |  |
| ° Pre Kidney Tx | 1 |  |  | 1 |  |
| ° Post Kidney Tx | 1.26 | 0.73-2.18 |  | 1.50 | 0.84-2.70 |
| Localisation imaging |  |  |  |  |  |
| ° Yes | 1 |  |  | 1 |  |
| ° No | 0.67 | 0.47-0.96 |  | 0.72 | 0.48-1.06 |
| Duration of surgery, minutes |  |  |  |  |  |
| ° <71 | 1 |  |  | 1 |  |
| ° 71-110 | 0.77 | 0.50-1.17 |  | 0.97 | 0.61-1.56 |
| ° >110 | 0.98 | 0.65-1.47 |  | 1.23 | 0.72-2.09 |
| Type of surgery |  |  |  |  |  |
| ° Less than subtotal | 1 |  |  | 1 |  |
| ° Subtotal | 1.10 | 0.53-2.29 |  | 0.96 | 0.45-2.06 |
| ° Total | 2.28 | 0.62-8.31 |  | 1.98 | 0.52-7.48 |
| ° Total + PT transplant | 2.33 | 1.10-4.92 |  | 1.82 | 0.82-4.02 |
| ° Re-operative | 1.77 | 0.78-4.05 |  | 1.97 | 0.82-4.75 |
| Thymus operation |  |  |  |  |  |
| ° No | 1 |  |  | 1 |  |
| ° Biopsy | 0.48 | 0.06-3.69 |  | 0.72 | 0.09-5.91 |
| ° Resection | 1.01 | 0.70-1.47 |  | 1.29 | 0.83-1.99 |
| Thyroid operation |  |  |  |  |  |
| ° No | 1 |  |  | 1 |  |
| ° Total thyroidectomy | 0.88 | 0.34-2.29 |  | 0.98 | 0.35-2.69 |
| ° Lobectomy | 0.96 | 0.54-1.71 |  | 0.92 | 0.50-1.72 |
| ° Less than lobectomy | 0.94 | 0.36-2.44 |  | 0.95 | 0.35-2.63 |
| Frozen section |  |  |  |  |  |
| ° No | 1 |  |  | 1 |  |
| ° Correct | 1.03 | 0.69-1.55 |  | 0.87 | 0.55-1.12 |
| ° Misleading | N/A |  |  | N/A |  |
| ioPTH |  |  |  |  |  |
| ° No | 1 |  |  | 1 |  |
| ° Correct | 0.83 | 0.57-1.20 |  | 0.76 | 0.51-1.12 |
| ° Misleading | 0.98 | 0.14-0.21 |  | 1.10 | 0.43-2.81 |

CI: confidence interval - ioPTH: intra-operative parathyroid hormone measurement – OR: odds ratio - PTH: parathyroid hormone – PT: parathyroid - Tx: transplant

SUPPLEMENTARY TABLE 3. PATIENT CHARACTERISTICS – SUBGROUP ANALYSIS COMPARING FIRST TIME SURGERY WITH TOTAL PARATHYROIDECTOMY VERSUS SUBTOTAL PARATHYROIDECTOMY

|  | All | Subtotal PTx | Total PTx |  |
| --- | --- | --- | --- | --- |
|  | N = 643 | N = 395 | N = 248 | *P-value* |
| SEX |  |  |  |  |
| ° Female | **316 (49.1)** | **181 (45.8)** | **135 (54.4)** | *0.041* |
| ° Male | **445 (51.8)** | **69 (51.1)** | **70 (40,9)** |  |
| AGE (years) | **52 (41-60)** | **50 (40-60)** | **53 (43-61)** | *0.090* |
| BIOCHEMISTRY |  |  |  |  |
| ° S-calcium Total (mmol/L) | **2.29 (2.12-2.45)** | **2.27 (2.12-2.44)** | **2.31 (2.14-2.48)** | *0.184* |
| ° S-PTH |  |  |  |  |
| - >20 times above normal range | **288 (44.9)** | **159 (40.0)** | **129 (52.2)** | *0.010* |
| - 1-5 times above normal range | **25 (3.9)** | **17 (4.3)** | **8 (3.2)** |  |
| - 6-10 times above normal range | **91 (14.2)** | **66 (16.8)** | **25 (10.1)** |  |
| - 11-20 times above normal range | **232 (36.2)** | **150 (38.1)** | **82 (33.2)** |  |
| - Within normal range | **4 (0.6)** | **1 (0.3)** | **3 (1.2)** |  |
| - Not determined | **1 (0.2)** | **1 (0.3)** | **0 (0.0)** |  |
| LOCALISATION EXAMINATION |  |  |  |  |
| ° No | **225 (35.0)** | **158 (40.0)** | **67 (27.0)** | *<0.001* |
| ° Yes | **403 (62.7)** | **222 (56.2)** | **181 (73.0)** |  |
| *° Missing* | *15 (2.3)* | *15 (3.8)* | *0 (0.0)* |  |
| OPERATION TIME (minutes) | **60 (40-99)** | **65 (45-105)** | **50 (35-92)** | *<0.001* |
| PARATHYROID REIMPLANTATION |  |  |  |  |
| ° No | **382 (59.4)** | **382 (96.7)** | **0 (0.0)** | *<0.001* |
| ° Yes | **261 (40.6)** | **13 (3.3)** | **248 (100)** |  |
| NUMBER OF IDENTIFIED PARATHYROIDS |  |  |  |  |
| ° 3 | **2 (0.3)** | **2 (0.5)** | **0 (0.0)** | *0.866* |
| ° 4 | **633 (98.4)** | **388 (98.2)** | **245 (98.8)** |  |
| ° 5 | **8 (1.2)** | **5 (1.3)** | **3 (1.2)** |  |
| THYMUS OPERATION |  |  |  |  |
| ° No | **490 (76.2)** | **279 (70.6)** | **211 (85.1)** | *<0.001* |
| ° Yes | **153 (23.8)** | **116 (29.4)** | **37 (14.9)** |  |
| PEROPERATIVE RLN DAMAGE |  |  |  |  |
| ° Bilaterally | **3 (0.5)** | **1 (0.3)** | **2 (0.8)** | *0.069* |
| ° Left | **3 (0.5)** | **3 (0.8)** | **0 (0.0)** |  |
| ° No Damage | **632 (98.3)** | **390 (98.7)** | **2442 (97.6)** |  |
| ° Right | **5 (0.8)** | **1 (0.3)** | **4 (1.6)** |  |
| FROZEN SECTION |  |  |  |  |
| ° Yes | **125 (19.4)** | **63 (15.9)** | **62 (25.0)** | *0.007* |
| ° No | **518 (80.6)** | **332 (84.1)** | **186 (75.0)** |  |
| INTRAOPERATIVE PTH |  |  |  |  |
| ° Correct | **185 (28.8)** | **114 (28.9)** | **71 (28.6)** | *0.644* |
| ° False Positive | **1 (0.2)** | **1 (0.3)** | **0 (0.0)** |  |
| ° False Negative | **10 (1.6)** | **8 (2.0)** | **2 (0.8)** |  |
| ° Not Used | **447 (69.5)** | **272 (68.9)** | **175 (70.6)** |  |
| MORBIDITY |  |  |  |  |
| ° No | **590 (91.8)** | **367 (92.9)** | **223 (89.9)** | *0.232* |
| ° Yes | **53 (8.2)** | **28 (7.1)** | **25 (10.1)** |  |
| REVISION FOR BLEEDING |  |  |  |  |
| ° No | **624 (97.0)** | **379 (95.9)** | **245 (98.8)** | *0.038* |
| ° Yes | **10 (1.6)** | **7 (1.8)** | **3 (1.2)** |  |
| *° Missing* | **9 (1.4)** | **9 (2.3)** | **0 (0.0)** |  |
| TIME TO DISCHARGE (days) | **4 (2-6)** | **3 (2-5)** | **5 (3-6)** | *<0.001* |
| WOUND INFECTION |  |  |  |  |
| ° No | **627 (97.5)** | **381 (96.5)** | **246 (99.2)** | *0.065* |
| ° Yes | **4 (0.6)** | **3 (0.8)** | **1 (0.4)** |  |
| *° Missing* | **12 (1.9)** | **11 (2.8)** | **1 (0.4)** |  |

PTH: parathyroid hormone - PTx: parathyroidectomy – S-PTH: serum parathyroid hormone
